# Orthostatic Intolerance Is Independent of the Degree of Autonomic Cardiovascular Adaptation after 60 Days of Head-Down Bed Rest

**DOI:** 10.1155/2015/896372

**Published:** 2015-09-03

**Authors:** Jiexin Liu, Yongzhi Li, Bart Verheyden, Zhanghuang Chen, Jingyu Wang, Yinghui Li, André E. Aubert, Ming Yuan

**Affiliations:** ^1^Department of Cardiology, Beijing Friendship Hospital, China Capital Medical University, Beijing 100050, China; ^2^Department of Cardiology, University Hospital Gasthuisberg, KU Leuven, 3000 Leuven, Belgium; ^3^State Key Laboratory of Space Medicine Fundamentals and Application, China Astronaut Research and Training Center, Beijing 100094, China

## Abstract

Spaceflight and head-down bed rest (HDBR) can induce the orthostatic intolerance (OI); the mechanisms remain to be clarified. The aim of this study was to determine whether or not OI after HDBR relates to the degree of autonomic cardiovascular adaptation. Fourteen volunteers were enrolled for 60 days of HDBR. A head-up tilt test (HUTT) was performed before and after HDBR. Our data revealed that, in all nonfainters, there was a progressive increase in heart rate over the course of HDBR, which remained higher until 12 days of recovery. The mean arterial pressure gradually increased until day 56 of HDBR and returned to baseline after 12 days of recovery. Respiratory sinus arrhythmia and baroreflex sensitivity decreased during HDBR and remained suppressed until 12 days of recovery. Low-frequency power of systolic arterial pressure increased during HDBR and remained elevated during recovery. Three subjects fainted during the HUTT after HDBR, in which systemic vascular resistance did not increase and remained lower until syncope. None of the circulatory patterns significantly differed between the fainters and the nonfainters at any time point. In conclusion, our data indicate that the impaired orthostatic tolerance after HDBR could not be distinguished by estimation of normal hemodynamic and/or neurocardiac data.

## 1. Introduction

The lack of gravitational stress during spaceflight is associated with many adaptations in cardiovascular structure as well as neurohumoral control circuits [1–7]. One important negative manifestation is postspaceflight orthostatic intolerance (OI) [8], which is a current operational problem for many astronauts. Due to limitations related to human spaceflight, such as high cost, limited access, small number of subjects, and limited crew time, head-down bed rest (HDBR) has been used to investigate the physiologic effects of spaceflight as a microgravity simulation model [9].

Cardiovascular adaptations to HDBR have been studied over the past 20 years. During the first hours of HDBR, body fluid shifts from the lower to the upper part of the body, resulting in a transient increase in thoracic plasma volume [10, 11]. This expanded plasma volume simulates central volume carotid, aortic, and cardiac receptors, induces an increase in diuresis and natriuresis as well as a decrease in plasma volume, and restores central blood volume to baseline levels after a couple of days [12–14]. Moreover, it has been reported that HDBR leads to a decrease in vagal control of the cardiac system and an increase in vasomotor sympathetic tone as well as a decrease in baroreflex sensitivity (BRS) [12, 15–18]. Similar to what occurs after spaceflight, these adaptations facilitate the occurrence of postural tachycardia with a variable degree of OI after HDBR [9].

A number of studies have focused on the mechanism of postspaceflight or post-HDBR orthostatic intolerance. Most of the studies were conducted with postural stress, such as the head-up tilt test (HUTT). Nevertheless, whether or not OI relates to the degree of autonomic cardiovascular adaptations is uncertain. If it does not relate to the degree of autonomic cardiovascular adaptations, what are the determinants of this phenomenon? In addition, another attractive and practical question is to determine who will have poor orthostatic tolerance after microgravity or microgravity simulation; that is, can postspaceflight or post-HDBR OI be predicted by the responses to a normal HUTT?

The purpose of this study was to test the hypothesis that fainters (subjects with a positive orthostatic stress test after HDBR) will have lower vagal cardiac control, a lower arterial baroreflex, and higher sympathetic activity after prolonged HDBR compared to nonfainters (subjects with a negative orthostatic stress test after HDBR) by detecting the differences in hemodynamic and cardiovascular autonomic control as well as arterial baroreflex function in fainters and nonfainters.

## 2. Materials and Methods

### 2.1. Study Population

Fourteen healthy male volunteers of Asian descent (age, 30 ± 1 years; height, 169 ± 1 cm; weight, 62 ± 1 kg) were enrolled in this 60-day HDBR study. All subjects spent 90 days in the Bed Rest Study Lab in the China Astronaut Research and Training Center (ACC; Beijing, China) as follows: 15-day pretest period for baseline data collection (BDC); 60-day −6° head-down tilt (HDT) period; and 15-day recovery period (*R*). The protocol conformed to the Helsinki Declaration and was approved by the Ethics Committee of the China Astronaut Research and Training Center. Each subject was thoroughly briefed on the experimental procedures prior to giving written consent. All subjects were in excellent health without a history of chronic or recent acute illnesses. The dietary intake was 2600–2800 cal/day (90–100 g/day of protein, 85–95 g/day of fat, and 390–430 g/day of carbohydrate). None of the subjects participated in regular athletics. Cigarette smoking, alcohol consumption, and caffeine-containing drinks were not allowed during the study.

### 2.2. Study Protocol

On the first day of bed rest, after showering and breakfast, the subjects moved from standing upright to a −6° head-down position. Three or four subjects were located in each observation room. The room temperature was 23°C–25°C. During the bed rest period, subjects were required to maintain the head-down position with their back and buttocks in contact with the bed; the subjects were permitted to use the bathroom for defecation once a day (5–10 min). During their free time, the subjects could watch television, listen to music, read books or newspapers, and interact with others. Compliance at night time was assured by camera surveillance.

Data collection was performed before (–BDC 14), during (HDT 2, 7, 21, 41, and 56), and after (*R* + 6 and *R* + 12) bed rest. Recording took place in the morning before 12:00 in a temperature-controlled room (24°C). We chose a 60-day period because it is long enough to eliminate acute transient phenomena at the onset of HDBR and not too long to burden the subjects.

The protocol started with the subjects resting quietly in the supine (before and after HDBR) or in head-down position (during HDBR) with comfortable, uncontrolled respirations for 10 min as a baseline recording. Then, the subjects were instructed to pace their breathing to an audio stimulus. Controlled breathing was held for 3 min, during which respiratory sequences were evenly spaced in time at a preset rate of 12 breaths min^−1^ or 0.2 Hz [19].

A HUTT was performed twice at 75° before (at BDC 4 day) and after bed rest (*R* + 0). After a period of 10 min in the supine position, volunteers were tilted to the 75° head-up tilt position for a maximum duration of 20 min. Immediately after fainting or completing the entire tilt duration, that is, 20 min, the subjects were tilted back to the supine position for a 10 min recovery.

During the HUTT, syncope was defined as the loss of consciousness accompanied by severe hypotension and/or bradycardia (systolic blood pressure <70 mmHg; heart rate (HR) decrease >15 beats/min). At the time of impending syncope, typical symptoms and signs included lightheadedness, nausea, blurred vision, pallor, and sweating.

Volunteers were regarded as nonfainters or fainters according to the HUTT results after 60 days of HDBR. The nonfainters were defined as subjects who completed HUTT without fainting; in contrast, the fainters were defined as subjects who had the test aborted due to syncope.

### 2.3. Data Acquisition and Analysis

Noninvasive beat-to-beat blood pressure (BP) was measured with an infrared finger photoplethysmograph (Finometer Blood Pressure Monitor; TNO-TPD Biomedical Instrumentation, Amsterdam, Netherlands). The hand was held at heart level to avoid hydrostatic pressure differences. The measured signal was analog-to-digital converted at 1000 Hz and stored on a hard disk for offline analysis. Systolic arterial pressure and diastolic arterial pressure were derived from the arterial pressure waveform. Mean arterial pressure (MAP) was the true integral of the arterial pressure wave over one beat divided by the corresponding beat interval. Pulse pressure (PP) was defined as the difference between systolic pressure and diastolic pressure. HR was computed from ECG recording and expressed as beats per minute.

During the 3 min paced breathing protocol, beat-to-beat SAP and the RR interval (RRI) time series were constructed for frequency analysis. First, two linear filters (SD filter and 20% mean values) were applied to correct for data points outside a limit interval [20]. The resulting beat-to-beat hemodynamic time series was interpolated using a cubic-spline approximation and resampled at 2 Hz to construct equidistant time series. A sliding window of 128 sec (256 samples) was applied with 16 sec increments. This process resulted in four segments of data in each time series. The DC component was removed by subtracting the mean value, and a Hanning window was applied. A nonparametric “run test” of means and mean square values was used to validate the stationarity of data within 5% of the confidence limits [20]. In the resulting time windows, power spectral density was averaged using fast Fourier transformation. The spectral resolution for all estimates equaled 0.0078 Hz. Respiratory powers were expressed as the area under the spectrum from 0.18 to 0.22 Hz and used as a marker of respiratory sinus arrhythmia (RSA). A second spontaneous rhythm occurring over an approximate 10 sec cycle and resulting in a low-frequency band (0.04–0.15 Hz) was obtained as well for SAP variability [21]. Power spectral units for RRI and SAP fluctuations were squared amplitudes.

During the 10 min baseline recording time domain analysis of spontaneous BRS was performed using the cross-correlation method [22]. In a 10 sec window, the correlation and regression slope between SAP and RRI were computed. Delays of 0–5 sec increments in RRI were computed, and the delay with the highest positive coefficient of correlation was selected (tau). The slope between SAP and RRI was recorded as BRS estimate if the correlation was significant at a *P* = 0.01. The number of significant BRS estimates was also calculated and expressed per minute. Thus, the following three complementary aspects of arterial baroreflex function were assessed: (1) BRS, which provides qualitative information; (2) number of BRS estimates, which provides quantitative information; and (3) tau, which provides temporal information on cardiac baroreflex control.

Orthostatic intolerance before and after HDBR was tested with HUTT. Continuous changes in stroke volume (SV) were estimated by modeling flow from the arterial pressure waveform (Modelflow; TNO Biomedical Instrumentation) expressed in mL [23]. Cardiac output (CO; expressed in L/min) was the product of the estimated SV and HR, and systemic vascular resistance (SVR; expressed in mmHg·s/mL [also referred to as medical units or MUs]) was the MAP at the heart level divided by the computed CO. The Modelflow method computes aortic flow from the finger arterial pressure by simulating a nonlinear model of the aortic input impedance [23]. This pulse wave analysis method corrects for individual differences in age, gender, height, and weight [24], thus permitting group average data to be examined accurately in subjects without structural or functional heart disease [25, 26]. Previous head-up tilt experiments have demonstrated good agreement between Modelflow and standard CO estimates, such as inert gas rebreathing [27], thermodilution [28], and Doppler ultrasound [29]. Under the adverse circumstances of very low arterial BPs, Modelflow computations have also been shown to be accurate [30].

### 2.4. Statistical Analysis

Statistical analysis was performed with SPSS (version 13.0 for Windows; Scientific Packages for Social Sciences, Inc., Chicago, IL, USA) and Prism 5.0 (GraphPad, San Diego, CA, USA). Data are given as the median (25, 75 percentiles) or mean ± SD. Spectral data were logarithmically transformed if needed to approximate normal distributions. The evolutions of hemodynamic and spectral and baroreflex indices before, during, and after HDBR were analyzed across conditions using one-way repeated measures ANOVA if data passed the D'Agostino and Pearson omnibus normality test. Simple contrast analysis was conducted to evaluate changes at each time point as compared to baseline by Bonferroni's multiple comparison test. If any data didn't pass the normality test, they were analyzed by Kruskal-Wallis test and then performed Dunn's test for multiple comparison. During the HUTT before and after HDBR, hemodynamic parameters in the nonfainters were evaluated and compared with two-way repeated measures ANOVA. *P* values < 0.05 were considered statistically significant.

## 3. Results

All of the subjects completed the study. No volunteer fainted during the HUTT before HDBR; three volunteers were regarded as fainters because of the positive HUTT after HDBR, and the others (*n* = 11) were regarded as nonfainters. Before HDBR, there were no differences in age, weight, and height between the nonfainters (age, 30 ± 4 years; weight, 60 ± 4 kg; height, 169 ± 4 cm) and the fainters ((A): age, 27 years; weight, 63 kg; height, 173 cm; (B): age, 29 years; weight, 58 kg; height, 170 cm; (C): age, 39 years; weight, 66 kg; height, 173 cm). After HDBR, the weight increased in the two groups as follows: nonfainters, 63 ± 5 kg (*P* = 0.008, compared to before HDBR), and fainters ((A), 67 kg; (B), 64 kg, and (C), 70 kg).

### 3.1. Evolutions in Circulatory Control

**Figure 1:**
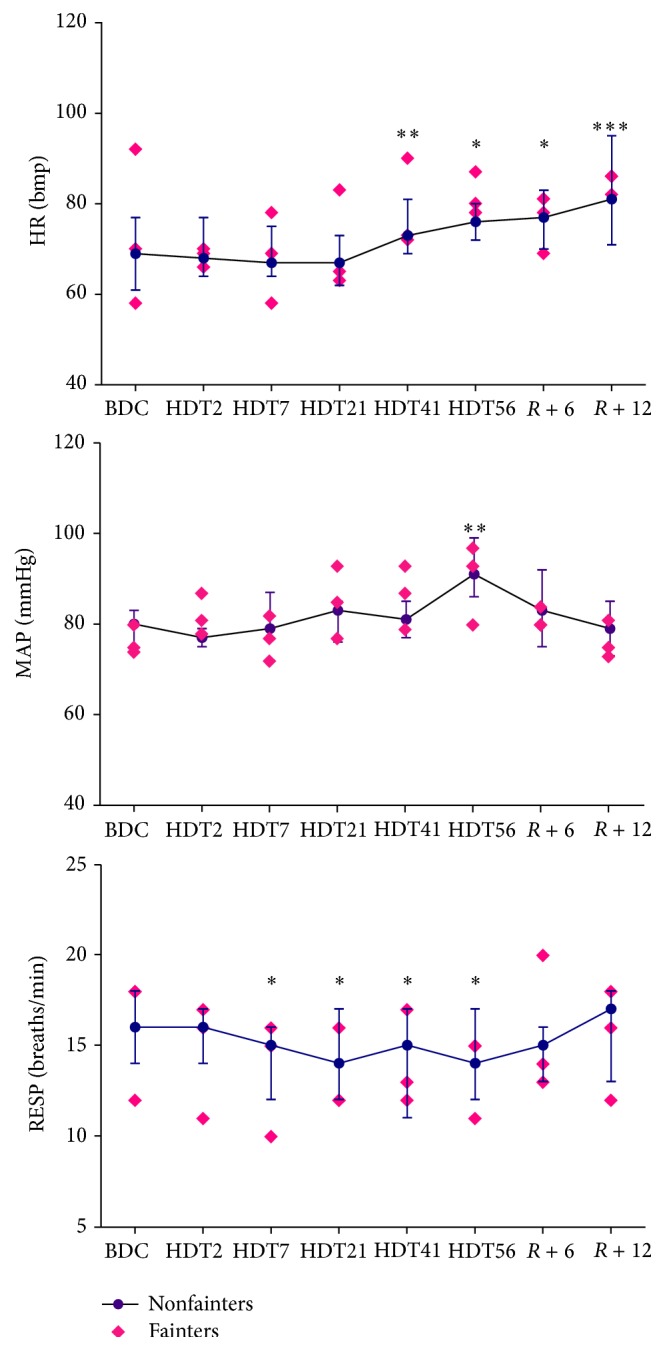
Changes in heart rate (HR), mean arterial pressure (MAP), and respiration frequency (RESP) during HDBR in the fainters and the nonfainters. Nonfainters, the median values [25th, 75th percentiles] are shown as dots, *n* = 11; fainters, the absolute individuals values are shown as diamonds, *n* = 3; BDC, 14 days before head-down bed rest; HDT2, the 2nd day of bed rest; HDT7, the 7th day of bed rest; HDT21, the 21st day of bed rest; HDT41, the 41st day of bed rest; HDT56, the 56th day of bed rest; *R* + 6, the 6th day during recovery; *R* + 12, the 12th day during recovery. ^*∗*^ *P* < 0.05, ^*∗∗*^ *P* < 0.01, and ^*∗∗∗*^ *P* < 0.001 compared with baseline before HDBR.

**Figure 2:**
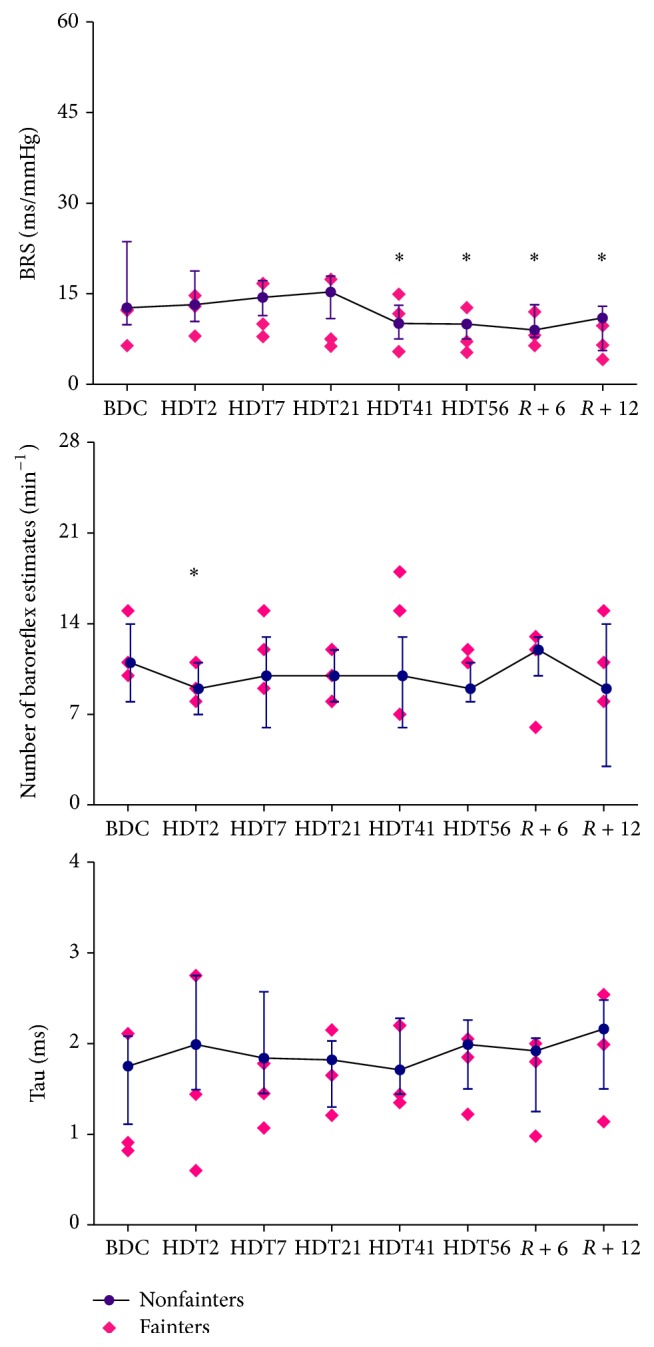
Changes in baroreflex sensitivity (BRS), number of baroreflex estimates per minute, and tau (optimal time delays) during HDBR in the fainters and the nonfainters. Nonfainters, the median values [25th, 75th percentiles] are shown as dots, *n* = 11; fainters, the absolute individuals values are shown as diamonds, *n* = 3; BDC, 14 days before head-down bed rest; HDT2, the 2nd day of bed rest; HDT7, the 7th day of bed rest; HDT21, the 21st day of bed rest; HDT41, the 41st day of bed rest; HDT56, the 56th day of bed rest; *R* + 6, the 6th day during recovery; *R* + 12, the 12th day during recovery. ^*∗*^ *P* < 0.05 compared with baseline before HDBR.

**Figure 3:**
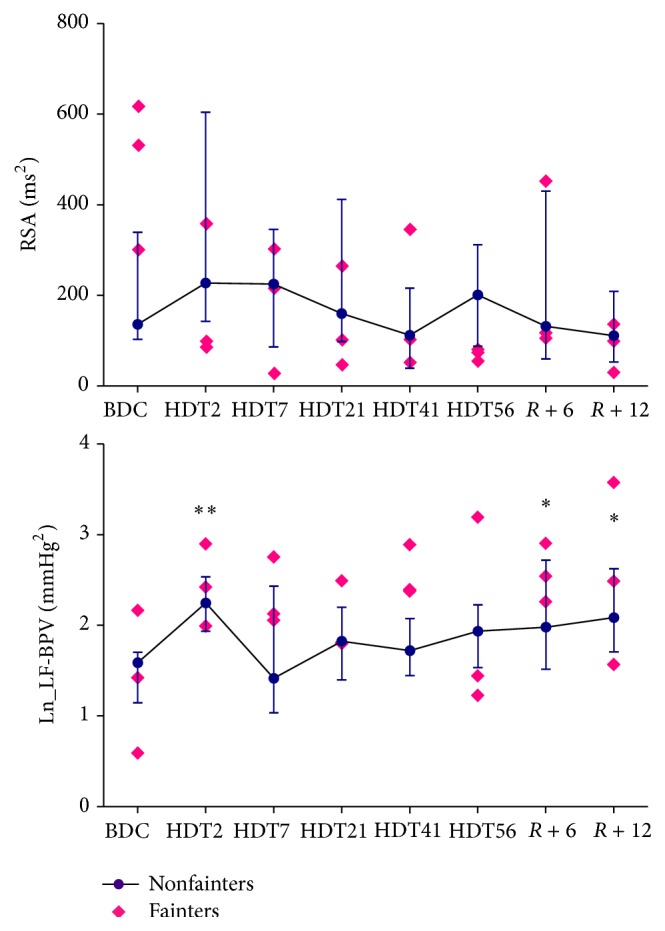
Changes in respiratory sinus arrhythmia (RSA) and LF power of BPV during HDBR in the fainters and the nonfainters. Nonfainters, the median values [25th, 75th percentiles] are shown as dots *n* = 11; fainters. the absolute individuals values are shown as diamonds, *n* = 3; BDC. 14 days before head-down bed rest; HDT2. the 2nd day of bed rest; HDT7. the 7th day of bed rest; HDT21. the 21st day of bed rest; HDT41. the 41st day of bed rest; HDT56. the 56th day of bed rest; *R* + 6. the 6th day during recovery; *R* + 12. the 12th day during recovery. ^*∗*^ *P* < 0.05 and ^*∗∗*^ *P* < 0.01 compared with controlled breathing data before HDBR.

Figure 1 shows the changes in HR, MAP, and respiratory frequency before, during, and after HDBR. On average, in all nonfainters (*n* = 11), there was a progressive rise in HR until day 56 of HDT (from 69 beats/min [61, 77 beats/min] before HDBR to 73 beats/min [69, 81 beats/min] at HDT41, *P* < 0.01). The HR remained significantly higher until day 12 of recovery. The MAP increased gradually until day 56 of HDT (from 80 mmHg [75, 83 mmHg] before HDBR to 91 mmHg [86, 99 mmHg] at HDT56, *P* < 0.001) and then returned to baseline after 12 days of recovery. The respiration frequency decreased during HDBR (from 16 min^−1^ [14, 18 min^−1^] before HDBR to 14 min^−1^ [12, 17 min^−1^] at HDT56, *P* < 0.05) and returned to baseline during the recovery. Evolutions of BRS, the number of baroreflex estimates, and tau before, during, and after HDBR are shown in Figure 2. In all nonfainters, BRS significantly decreased at HDT56 (from 12.7 ms/mmHg [9.9, 23.6 ms/mmHg] before HDBR to 10.1 ms/mmHg [7.5, 13.1 ms/mmHg] at HDT41, *P* = 0.0241) and remained suppressed until R12. The number of baroreflex estimates per min decreased on HDT2 (from 11 min^−1^ [8, 14 min^−1^] before HDBR to 9.0 min^−1^ [7, 11 min^−1^] at HDT2, *P* = 0.033) and then gradually recovered to baseline after 56 days of HDBR. Tau remained stable during the HDBR and during recovery. The alterations in RSA and low-frequency power of SAP are shown in Figure 3. In nonfainters, RSA fluctuated during HDBR and returned to baseline during the recovery. Low-frequency power of SAP significantly increased on HDT2 (from 1.592 mmHg^2^ [1.148, 1.705 mmHg^2^] before HDBR to 2.25 mmHg^2^ [1.939, 2.537 mmHg^2^] at HDT2, *P* = 0.0095) and showed a tendency to be increased until HDT56. A further increase in the low-frequency power of SAP was observed during the recovery (1.948 mmHg^2^ [1.519, 2.723 mmHg^2^] at day R6, *P* = 0.015 and 2.089 mmHg^2^ [1.711, 2.629 mmHg^2^] at day R12, *P* = 0.0427 compared to data from controlled breathing before HDBR). None of the circulatory adaptations to HDBR significantly differed between the fainters and the nonfainters at any time point (Figures 1–3).

### 3.2. Orthostatic Circulatory Control

**Figure 4:**
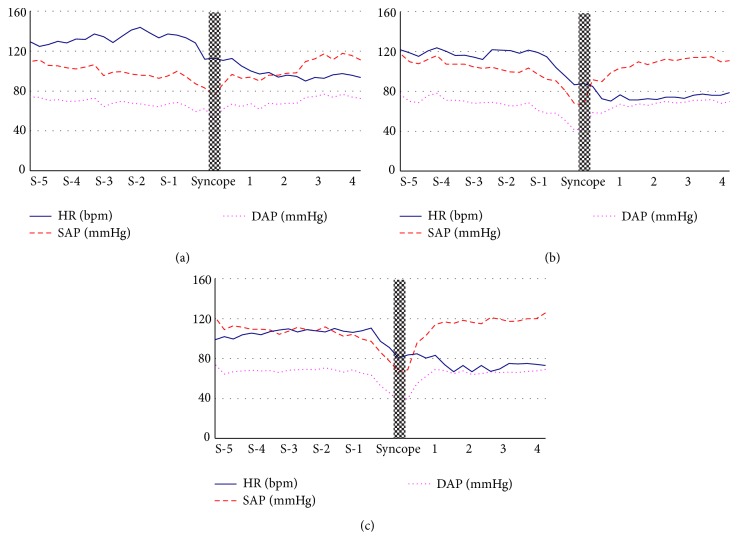
Heart rate (HR), systolic blood pressure (SBP), and diastolic arterial pressure (DAP) of 3 fainters (a, b, and c) 5 minutes before and 4 minutes after syncope during the positive HUTT after HDBR. Heart rate and blood pressure (BP) decreased together before the syncopal episode.

In the positive HUTT (*n* = 3), the time range from the start of tilt to the syncopal episode was from 6.8 to 12.0 min. The changes in HR, SAP, and DAP of the three fainters 5 min before and 4 min after the syncopal episodes during HUTT are shown in Figure 4. According to the VASIS classification of the European Society of Cardiology (ESC) [31], all fainters were of the mixed type (a fall in BP comes first, followed by a decrease in HR, without marked bradycardia or asystole). The drops in HR and BP started 1-2 minutes before the syncopal episode; the BP recovered quickly 2 min after tilting back to the supine position.

**Figure 5:**
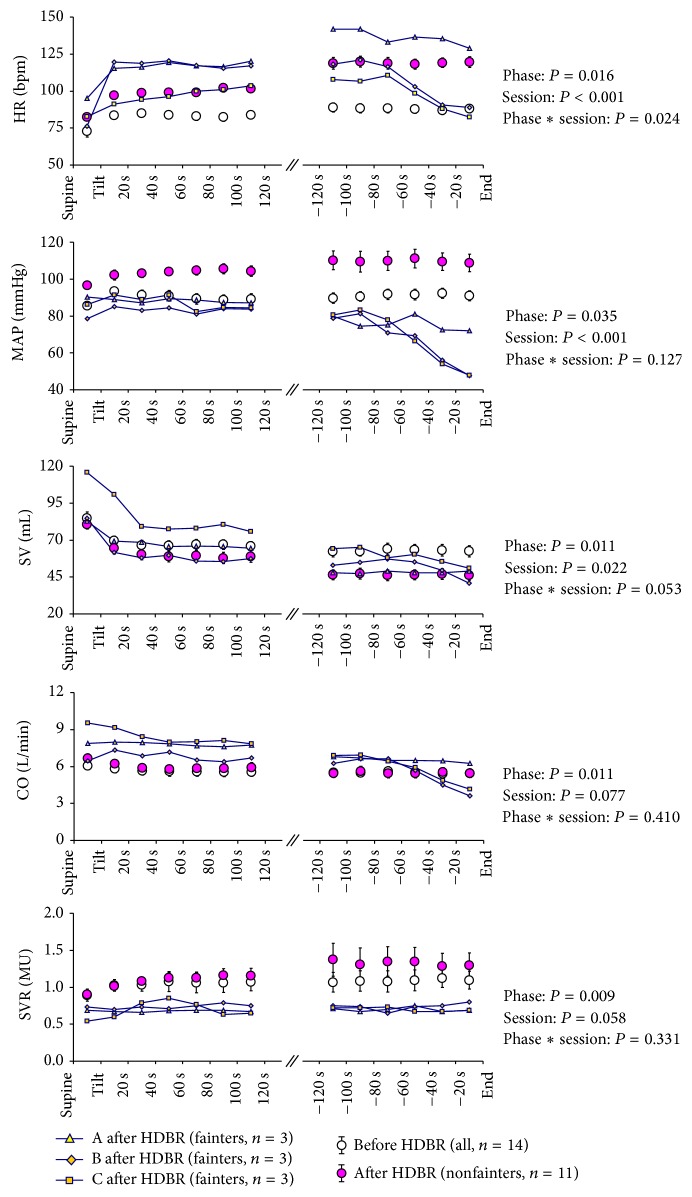
Changes in estimated heart rate (HR), mean arterial pressure (MAP), stroke volume (SV), cardiac output (CO), and systemic vascular resistance (SVR) from supine until the end of head-up tilt testing. Values in the supine position during the first and last 2 minutes of the tilt are presented as averages at 20-second intervals. In the nonfainters (*n* = 11), phase indicates the different moment during HUTT, and session includes two parts (pre-HDBR and post-HDBR).

Hemodynamic responses to tilting before and after HDBR are shown in Figure 5. Before HDBR, the responses to tilting were similar in all parameters in all subjects not only during the first 2 min immediately after the tilt but also during the last 2 min at the end of tilt testing. HR, MAP, and SVR significantly increased after tilting and remained higher until the end of tilting; SV and CO significantly decreased after tilting and remained lower until the end of HUTT. After HDBR, in the nonfainters (*n* = 11) at baseline in the supine position, hemodynamic parameters remained at the same level compared to pre-HDBR, except that there was a significant rise in MAP (from 84 mmHg before HDBR to 97 mmHg after HDBR, *P* = 0.003). Immediately after tilting, the HR and MAP significantly increased. These increases were more pronounced after HDBR (*P* < 0.001 in both HR and MAP); SV markedly decreased compared with a larger increase in SVR. Compared to the HUTT before HDBR, the decrease in SV was larger (*P* = 0.022) and the increase in SVR also showed a tendency to be increased (*P* = 0.058). CO significantly decreased similar to the previous one. At the end of tilt testing, HR increased further (*P* = 0.024); the MAP remained at the higher level (*P* = 0.127) and the SVR tended to increase slightly (*P* = 0.331), compensated by a further decrease in SV (*P* = 0.053) simultaneously. CO did not change compared to the beginning of tilting. In the three fainters, immediately after tilting, HR increased more in the two fainters compared to the nonfainters, but the MAP did not increase. The SV was significantly decreased in all fainters and, due to the larger increase in HR, CO seemed to be higher compared to the nonfainters. SVR was unchanged after tilting. Before a syncopal episode, SVR remained at a low level in all fainters. A steeper drop in the HR, MAP, SV, and CO was observed in two of the three fainters (Figure 5).

## 4. Discussion

In the current study cardiovascular and respiratory control was evaluated in 14 healthy volunteers before, during, and after a 60-day HDBR eliciting cardiovascular adaptations. This study focused on the possible influence of hemodynamic parameters altering autonomic cardiovascular control after HDBR. The main findings of the present investigation were twofold. First, the evolution in hemodynamic and respiratory parameters during long-term HDBR was similar in fainters and nonfainters; that is, OI after microgravity simulation was not predicted by normal estimation of hemodynamic and/or neurocardiac data. Second, in agreement with our previous findings in vasovagal syncope patients [32, 33], we found that prior to syncope there was a steep fall in CO in all fainters that was insufficiently compensated for by SVR.

During the past 20 years, HDBR has proven to be useful as a simulation model for space physiology studies on the ground. A number of previous studies have focused on cardiovascular regulation of the autonomic nervous system in bed rest studies. Most studies have reported a decrease in HRV and parasympathetic indices [16–18], implying a chronic cardiovascular adaptation to HDBR. In agreement with the European Space Agency, French Space Agency, and National Space Development Agency of Japan (ESA/CNES/NASDA) clinical report, which found a progressive increase in resting HR in the second half of the study during a 90-day HDBR [9], we also found that HR was significantly increased after HDT41; specifically, respiratory sinus arrhythmia decreased, while BP and the low-frequency power of BPV continuously increased. These results are in agreement with Kamiya et al. [34] who showed an increase in vasomotor sympathetic tone, as determined from muscle sympathetic nerve activity on the tibial nerve with long-term HDBR (60 and 120 days). This suggests that long-term HDBR results in a reduction in vagal cardiac control and a rise in sympathetic activity. Moreover, in agreement with previous studies showing that the spontaneous baroreflex slope decreased during and/or after HDBR [12, 16–18, 35], we observed a gradual reduced BRS slope during HDBR, suggesting that the cardiac baroreflex mechanism is disturbed after a prolonged period of bed rest. In addition, we also found a transient decrease in the number of baroreflex estimates at the beginning of HDBR. It is likely that the decrease in the quantity of BRS is consistent with the previously reported transient increase in thoracic blood volume [10, 11] due to the initial fluid shift from the lower part to the upper part of the body, while the decrease in the quality of BRS is coincident with the previously reported subsequent decrease in plasma volume and the restoration of central blood volume to baseline levels in the latter part of bed rest [12–14]. This finding implies that the quantitative and qualitative changes in BRS are elicited by reversed hemodynamics.

Furthermore, in agreement with previous findings during spaceflight [19, 36], our study showed a decrease in respiration frequency during HDBR. Although this special phenomenon is difficult to explain with our current knowledge, it might be speculated that a decrease in respiration frequency might be related to a reduction in lung volume [37], tidal volume, pulmonary ventilation, and metabolic rate [36], which may be a physiologic response to reduced metabolic demand [19]; however, further studies are needed.

It has been shown that exposure to actual or simulated microgravity alters physiologic adaptive mechanisms and reduces exercise capacity and orthostatic tolerance [8, 38–40]. Head-up tilt testing was performed as orthostatic stress in this study. With our current experimental protocol, orthostatic tolerance was reduced in three volunteers after HDBR. Based on our existing data, we rejected our hypothesis that the fainters would have lower vagal cardiac control, a lower arterial baroreflex, and higher sympathetic activity after prolonged HDBR. In fact, we could not distinguish the hemodynamic and autonomic differences between the fainters and nonfainters during and after HDBR with our definition of fainting by HUTT. Thus, we infer that fainting after bed rest does not relate to the degree of neural cardiovascular changes induced by HDBR. A number of previous studies have used the response to an orthostatic stress test to evaluate mechanisms of OI; however, our data suggest that this may not be a good strategy for investigating the neural contribution to OI because the individual response to an orthostatic stress test does not necessarily appear to distinguish between subjects who are deconditioned and those who are not.

Unexpectedly, we found only three of 14 fainters, whereas at least seven of 14 would be expected as fainters according to the literature (99 of 172 resulting from 12 male-only studies [references in the review by Pavy-Le Traon et al. [9]]). A possible reason might be the daily use of the bathroom by the subjects; however, daily toilet visits cannot be viewed as a specific countermeasure, as the behavior of all parameters was in agreement with the results from most studies [12, 16–18, 34, 35]. Another possible reason might be attributed to water intake, which has a significant influence on OI. Unfortunately, the daily water intake was not controlled in this study; thus we cannot evaluate whether or not the low occurrence rate of OI was due to the uncontrolled fluid intake. Moreover, an increase in weight for the fainters can also be hypothesized, although no physiologic correlation has been shown in the literature.

In the three fainters, the tilt-induced syncope episode appears to be cardiac output-mediated, with a rapid drop in HR and SV, which begins several minutes prior to the occurrence of syncope, but without a marked fall in SVR. These cardiovascular responses are precisely similar to what we found in the vasovagal syncope patients [32, 41]; however, two major differences existed between the fainters and the nonfainters in hemodynamic changes during orthostatic stress. First, the MAP was higher in the nonfainters than in the fainters at the beginning of HUTT and was maintained until the end of HUTT. Second, the nonfainters had a similarly increased SVR response to tilting before and after bed rest as well as a further increase at the end of HUTT after bed rest; however, in the fainters, SVR was lower from the beginning of HUTT until the occurrence of syncope. A logical explanation for this phenomenon might be the fact that HDBR reduced vasomotor modulation in the fainters, resulting in an impaired vasoconstriction response to the increase in postural change, leading to a diminished cardiovascular reserve. In agreement with our results, but using a different study approach, Buckey et al. [8] showed that nine of 14 astronauts could not complete a 10 min postflight stand test that all were able to complete before flight, suggesting that the subjects who were able to complete the test had a higher vasoconstrictor response and maintained SVR, while the fainters did not. In a study of Butler et al. [40], it was speculated that the cause of the impaired orthostatic tolerance is a decreased tone in venous capacitance vessels. Using ultrasonography, Arbeille et al. [42] also demonstrated that the fainters showed a lack of leg vasoconstriction and higher calf vein distensibility during HUTT after a 60-day HDBR. Therefore, we suggest that the impaired vasomotor modulation might be a main reason for OI after spaceflight or HDBR. A recent study involving vasovagal syncope by Verheyden et al. [32] provided circumstantial evidence of this outcome. It was documented that orthostatic tolerance can be improved by increasing the amount of vasoconstriction after 6 weeks of continued standing training, which increased the vasoconstrictor reserve in vasovagal syncope patients [32]; however, further studies are still needed.

The present results suggest that prolonged HDBR results in a decreased BRS, which is similar to postspaceflight. Fritsch et al. [43] observed a functionally relevant impairment in BRS after missions of 4-5 days in space and a reduction of BRS after missions of 8–14 days [44]. Cooke et al. [45] reported a reduced vagal baroreflex gain after a 9-month long duration space mission; however, we suggest that the similar decrease in BRS could be due to different mechanisms, such as immobilization and inactivity in HDBR and lack of gravity stimulation during space missions.

Unlike HDBR, HR has been reported to be stable [4, 19] or decreased [3, 45–47] during spaceflight. Additionally, it is likely that the differences between HDBR and real spaceflight are due to the great environment dissimilarity. A reasonable interpretation of these differences is the fact that as a ground-based simulation model HDBR cannot eliminate the influence of gravity. During bed rest, hydrostatic pressure at the heart level is higher than that during the supine position as well as during the standing position. This enhances the preload of the heart as well as the afterload acting as a feedback mechanism to increase the HR. During spaceflight, however, several studies have demonstrated a decrease in central venous pressure (CVP) [48, 49] together with increased cardiac chamber volumes [48]. Additionally, the strains during microgravity related to tissue compressions created by gravity disappear, leading to vasodilatation. This is facilitated by a decreased pleural pressure, which increases the distensibility of the pulmonary vessels and the vasculature [50]. As a result, the preload might increase, but the afterload probably decreases in space.

## 5. Conclusion

In all subjects, HDBR resulted in a decreased vagal cardiac control, an increased sympathetic activity, and a decreased arterial baroreflex. Post-HDBR orthostatic hypotension elicited by tilt testing is cardiac output-mediated, in which the lack of vasoconstriction also appears as an important inducement. The impaired orthostatic tolerance after HDBR could not be distinguished by estimation of normal hemodynamic and/or neurocardiac data.
